# Non-Invasive Approach for Evaluation of Pulmonary Hypertension Using Extracellular Vesicle-Associated Small Non-Coding RNA

**DOI:** 10.3390/biom9110666

**Published:** 2019-10-29

**Authors:** Christoph Lipps, Philipp Northe, Ricardo Figueiredo, Manfred Rohde, Alexandra Brahmer, Eva-Maria Krämer-Albers, Christoph Liebetrau, Christoph B. Wiedenroth, Eckhard Mayer, Steffen D. Kriechbaum, Oliver Dörr, Holger Nef, Christian W. Hamm, Till Keller, Christian Troidl

**Affiliations:** 1 Medical Clinics I-Cardiology and Angiology, Justus-Liebig-University Giessen, 35392 Giessen, Germany; 2 Department of Cardiology, Kerckhoff Clinic GmbH, 61231 Bad Nauheim, Germany; 3 German Centre for Cardiovascular Research, Partner Site Rhine-Main, 61231 Bad Nauheim, Germany; 4 GenXPro GmbH, 60438 Frankfurt am Main, Germany; 5 Central Facility for Microscopy, Helmholtz Centre for Infection Research, 38124 Braunschweig, Germany; 6 Institute of Developmental Biology and Neurobiology, Biology of Extracellular Vesicles, Johannes Gutenberg-University, 55122 Mainz, Germany; 7 Department of Thoracic Surgery, Kerckhoff Clinic GmbH, 61231 Bad Nauheim, Germany

**Keywords:** biomarker, extracellular vesicles, small non-coding RNA, pulmonary disease, right heart dysfunction, chronic thromboembolic pulmonary hypertension

## Abstract

Extracellular vesicles are released by numerous cell types of the human body under physiological but also under pathophysiological conditions. They are important for cell–cell communication and carry specific signatures of peptides and RNAs. In this study, we aimed to determine whether extracellular vesicles isolated from patients with pulmonary hypertension show a disease specific signature of small non-coding RNAs and thus have the potential to serve as diagnostic and prognostic biomarkers. Extracellular vesicles were isolated from the serum of 23 patients with chronic thromboembolic pulmonary hypertension (CTEPH) and 23 controls using two individual methods: a column-based method or by precipitation. Extracellular vesicle- associated RNAs were analyzed by next-generation sequencing applying molecular barcoding, and differentially expressed small non-coding RNAs were validated by quantitative real-time polymerase chain reaction (qRT-PCR). We identified 18 microRNAs and 21 P-element induced wimpy testis (PIWI)-interacting RNAs (piRNAs) or piRNA clusters that were differentially expressed in CTEPH patients compared with controls. Bioinformatic analysis predicted a contribution of these piRNAs to the progression of cardiac and vascular remodeling. Expression levels of DQ593039 correlated with clinically meaningful parameters such as mean pulmonary arterial pressure, pulmonary vascular resistance, right ventricular systolic pressure, and levels of N-terminal pro-brain natriuretic peptide. Thus, we identified the extracellular vesicle- derived piRNA, DQ593039, as a potential biomarker for pulmonary hypertension and right heart disease.

## 1. Introduction

Pulmonary hypertension (PH) is characterized by high blood pressure in pulmonary arteries, making the right side of the heart work harder. PH is classified into five groups based upon etiology and mechanism. Chronic thromboembolic pulmonary hypertension (CTEPH) is a progressive pulmonary vascular disease that is classified as group IV in the current clinical classification of PH [1]. It is characterized by persistent thromboembolic obstruction of pulmonary arteries and progressive vascular remodeling that leads to an increased invasively measured mean pulmonary arterial pressure (mPAP) equal to or higher than 25 mmHg. The increase in pulmonary vascular resistance results in progressive vascular remodeling and right heart failure, limiting the life expectancy of the patients [2]. Patients with CTEPH have a poor prognosis if left untreated [3]. Thus, an early and accurate diagnosis of the underlying condition is required to apply the appropriate treatment strategy.

The potential diagnostic application of microRNAs (miRNAs) specific to individual diseases has been described in detail based on their pathophysiological profile [4,5,6]. Different expression levels of certain miRNAs have recently been shown in CTEPH patients compared with control individuals [7,8]. miRNAs belong to the group of small non-coding RNAs (sncRNAs), which are small regulatory molecules that influence basic biological functions in almost all cell types. In addition to miRNAs other sncRNAs have been studied within recent years. Thus, initial observations of differentially abundant circulating P-element induced wimpy testis (PIWI)-interacting RNAs (piRNAs) in serum have been reported [9,10]. piRNAs are small RNAs defined by their ability to specifically bind to PIWI proteins [11]. Evidence for the use of piRNAs as potential biomarkers, in addition to well-known miRNAs, has also been presented for complex lung diseases [12].

Circulating nucleic acids are often encapsulated into extracellular vesicles (EVs) that are continuously released from various cell types and that protect their contents from degradation. EVs are essential for the maintenance of intercellular communication and play a major role in the regulation of physiological and pathophysiological processes [13,14,15]. As sncRNAs are selectively packaged into EVs, their profile varies as a function of the pathological situation [16,17]. Therefore, the identification of specific EV-associated sncRNA profiles could make them useful as biomarkers [18,19].

In the present study, we compared the abundance of EV-associated sncRNAs in therapy-naïve CTEPH patients with that of healthy controls and identified an EV-associated piRNA that reflects the severity of the disease.

## 2. Materials and Methods

### 2.1. Sample Collection

Samples were used from 23 randomly selected CTEPH patients with mPAP ≥30 mmHg that were enrolled in an ongoing biomarker registry at the Kerckhoff Heart and Thorax Center (Bad Nauheim, Germany) [20]. This registry includes CTEPH patients referred to the center for scheduled pulmonary endarterectomy. As controls, 23 age- and sex-matched individuals enrolled in the ongoing BioProspective biomarker registry were used. This registry enrolls patients with suspected chronic coronary syndrome schedule for an invasive diagnostic procedure. For the present analyses only individuals without coronary artery disease, with preserved left ventricular function, and without known valvular disease were considered for matching. Table 1 provides clinical characteristics of these patients and controls.

Serum was collected at the time of study enrolment before any invasive or surgical procedure using EDTA as an anticoagulant followed by centrifugation at 3000× *g* for 10 min, and samples were then frozen at −80 °C until analysis. The approval of the local Ethics Committee at the University of Giessen was obtained for both cohorts used (approval numbers: 147/11 for the control cohort and 43/14 and 44/14 for the CTEPH cohort). All patients gave written, informed consent, including for the use and storage of serum and for future biomarker analysis.

### 2.2. Isolation of Extracellular Vesicles

EVs were isolated by two different procedures: first, a precipitation-based method using the total exosome isolation (TEI) reagent for serum (Invitrogen, Darmstadt, Germany) and second, a membrane affinity-based method, employing the ExoEasy Maxi Kit (Qiagen, Hilden, Germany) (ExoEasy). For isolation of EVs using the precipitation-based protocol, 1 mL patient serum was processed according to the manufacturer’s instructions. Serum was first centrifuged at 2000× *g* for 30 min to remove cells and debris. The supernatant was then mixed with 200 µL TEI reagent and incubated for 30 min at 4 °C. Following centrifugation at 10,000× *g* for 10 min at room temperature, the EV-containing pellet was resuspended in either 350 µL Qiazol (Qiagen, Hilden, Germany) for RNA isolation, 80 µL lysis buffer (WCE, see Western blotting) for downstream analysis of proteins, or 100 µL phosphate-buffered saline (PBS) for Nanoparticle tracking analysis (NTA).

For EV isolation based on membrane affinity, we made use of the ExoEasy Maxi Kit and the ExoRNeasy Midi Kit (Qiagen, Hilden Germany). This method utilized 2 mL of patient serum for Western blot analysis, 300 µL for quantitative real-time polymerase chain reaction (qRT-PCR) validation, and 100 µL for RNA sequencing.) Serum was centrifuged at 16,000× *g* for 10 min. For RNA sequencing the supernatant was filled up to 1 mL with PBS and filtered using a 0.8 µm filter. The filtered solution was mixed with binding buffer (XBP) and transferred to membrane affinity spin columns. The bound EVs were washed with washing buffer (XWP). For isolation, EVs were eluted using 2 mL elution buffer (XE buffer) followed by ultracentrifugation at 100,000× *g* for 2 h using polypropylene centrifuge tubes (Beckman Coulter, #326819) with a MLS-50 rotor. For downstream analysis of sncRNAs the EVs were lysed directly on the columns using 700 µL Qiazol.

### 2.3. RNA Isolation and Real-Time PCR

RNA was isolated using the miRNeasy Mini Kit (Qiagen, Hilden, Germany) or ExoRNeasy Midi Kit (Qiagen, Hilden, Germany) according to the manufacturer’s instructions. Cel-miR39 (Qiagen, Hilden, Germany) was added as spike-in control to each sample. Isolated RNA was transcribed using the TaqMan MicroRNA Reverse Transcription Kit (Thermo Scientific, Darmstadt, Germany) and application of three different RT primers from the respective TaqMan Small RNA Assays (Thermo Scientific, Darmstadt, Germany) for a single reaction (Suppl. Table S3 and Suppl. Table S4). Real-time PCR was performed using a CFX96 real-time PCR system (Bio-Rad Laboratories, Düsseldorf, Germany). Assays were performed in triplicate in a 20-μL reaction using TaqMan Universal PCR Master Mix II, no UNG (Thermo Scientific, Darmstadt, Germany). The amount of target RNA was standardized to the cel-miR39 spike-in control. Ratios were calculated by the ΔΔCT method [21] and the mean of controls was used for normalization.

### 2.4. RNA Sequencing

For RNA sequencing, RNA was isolated using the ExoRNeasy Midi kit with a starting volume of 100 µL serum (see above). sncRNA libraries were prepared using TrueQuant technology (GenXPro, Frankfurt am Main, Germany) for elimination of PCR bias. Briefly, modified 3′ and 5′ TrueQuant adapters were successively ligated to small RNA (<200 nt) using T4 RNA Ligase 2 and T4 RNA Ligase 1 (NEB, Frankfurt am Main, Germany), respectively. Adapter-ligated RNA was reverse transcribed with SuperScript III (Life Technologies, Darmstadt, Germany) and amplified by PCR with KAPA HiFi Hot-Start Polymerase (KAPA Biosystems, Wilmington, DE, USA). Amplified libraries were sequenced with HiSeq2000 (Illumina, San Diego, CA, USA). The data provided in the present publication have been deposited in NCBI’s Gene Expression Omnibus [22] and are accessible through GEO Series accession number GSE138107: (https://www.ncbi.nlm.nih.gov/geo/query/acc.cgi?acc=GSE138107).

### 2.5. PIWI-RNA Target Prediction and mRNA Functional Analysis

The targets of the differentially expressed piRNAs were identified using miRanda and the human transcriptome (hg38) [23,24]. Targets with a mean free energy of maximally 20 kcal/mol and very stringent score threshold of 190 were selected. The predicted mRNAs targeted by piRNA were used to perform pathway enrichment using DAVID Bioinformatics Resources 6.8 and the databases Gene Ontology, Reactome, and KEGG [25].

### 2.6. Nanoparticle Tracking Analysis

EV pellets were resuspended in particle-free PBS and analyzed at 23 °C (temperature controlled) using a Nanosight LM10 system (camera model Hamamatsu C11440-50B/A11893-02) equipped with a green laser (532 nm) (Malvern, Herrenberg, Germany). Samples were introduced for 30 s using an automatic syringe pump, and videos were captured at 25 frames/s. Individual particles were tracked using the Nanosight 2.3 software at camera level 13. For analysis, the conservative detection threshold was set to 8 and the minimum track length to automatic. The initial particle concentration in serum was calculated by using the respective dilution factors for each sample and the concentrations measured by nanoparticle tracking analysis.

### 2.7. Negative Staining of Isolated EVs for Transmission Electron Microscopy

Negative staining of isolated EVs fixed with 2% formaldehyde was performed on carbon support films modified according to Valentine et al. [26].

Thin carbon support films were prepared by sublimation of a carbon string onto a freshly cleaved mica surface (Bal-Tec SCD500, Liechtenstein). Fixed EVs were adsorbed onto the carbon film by floating a 3-mm piece of the carbon film on a 30-µL drop of fixed EVs for 30 s, washed in TE buffer (10 mM TRIS, 1 mM EDTA, pH 6.9), and subsequently washed in distilled water before transferring onto a drop of 2% (w/v) aqueous uranyl acetate, pH 5.0. The carbon piece with attached EVs was then taken up with a 300-mesh nickel grid and blotted dry onto a filter paper and subsequently air dried. Samples were examined with a TEM 910 transmission electron microscope (Carl Zeiss, Oberkochen, Germany) at an acceleration voltage of 80 kV. Images were taken at calibrated magnifications using a line replica and recorded digitally with a Slow-Scan CCD-Camera (ProScan, 1024 × 1024, Scheuring, Germany) with ITEM software (Olympus Soft Imaging Solutions, Münster, Germany). Contrast and brightness were adjusted with Adobe Photoshop 5.0.

### 2.8. Western Blotting

Western blot analysis was performed as previously described [27]. EV samples were lysed in WCE buffer (1 M Hepes, 5 M NaCl, 1 M Na_2_MoO_4_, 0.25% glycerin, 0.5 M ethylenediaminetetraacetic acid (EDTA), proteinase inhibitors leupeptin (0.005%), aprotinin (0.005%) and 0.5 mM phenylmethylsulfonyl fluoride (PMSF), with or without 1 mM Dithiothreitol (DTT)) on ice. After a 5 min incubation, 10% NP-40 was added followed by three bouts of sonication. After centrifugation at 14,000× *g* for 20 min, 4 °C, the total protein concentration in the supernatant was analyzed using the Bradford assay (Bio-Rad Laboratories, Düsseldorf, Germany).

For electrophoresis, 20 µg protein in each sample were reduced in 2× loading buffer (100 mM Tris pH 6.8, 4% sodium dodecyl sulfate (SDS), 0.2% Bromphenol Blue, 20% glycerol) and heated at 95 °C for 5 min. Samples were loaded onto a 4–20% Mini-Protean TGX stain-free protein gel (Bio-Rad, Düsseldorf, Germany) prior to transfer to a 0.45 µm polyvinylidene fluoride membrane using TransBlot (Bio-Rad, Düsseldorf, Germany). Membranes were blocked with 5% non-fat milk powder in PBS with Tween20 for 1 h at room temperature and then incubated with primary antibodies at the dilution specified by the provider at 4 °C overnight. Staining with secondary antibodies was performed for 1 h at room temperature. Detection was accomplished using the ChemiDoc MP Imaging System after developing the blots using the Clarity Western ECL Blotting Substrate Kit (Bio-Rad Laboratories, Düsseldorf, Germany).

Primary antibodies were purchased from Santa Cruz Biotechnology, USA (goat anti-TSG101 (1:1000), #sc-6037, mouse anti-CD81 (1:500), #sc-7637, mouse anti-ApoA1 (1:500), #sc-80551, mouse anti-ApoB (1:2000), #sc-13538), R&D Systems, USA (mouse-anti-albumin (1:3000), #MAB1455), and Abcam (rabbit anti-Syntenin (1:500), #ab133267). HRP-conjugated secondary antibodies were from Santa Cruz Biotechnology, USA (mouse anti-goat-igG-HRP (1:2000), #sc-2354, mouse anti-rabbit IgG- HRP (1:2000), #sc-2357), and from Cell Signaling, USA (horse anti-mouse IgG-HRP (1:2000), #7076).

### 2.9. Statistical Analysis

The sncRNA-seq data was quantified and tested for differential expression with omiRas [28]. Briefly, for each small RNA-seq library, data processing started with 3′ adapter clipping by a local alignment of the adapter sequence to each read. Subsequently, Illumina’s marked quality region was removed. Tags were mapped to the human genome (hg19) with Bowtie. The mapped tags were annotated with the help of various models of coding and non-coding RNAs retrieved from the University of California Santa Cruz (UCSC) table browser. Tags mapping to exonic regions of coding genes were excluded from further analysis. Non-coding RNAs were quantified in each library. Normalization and test for differential expression were calculated using the DESeq R/Bioconductor package [29]. Small RNAs with *p*-value <0.05 and |log2fc| >1 were considered as differentially expressed.

Real-time PCR data from CTEPH patients and control individuals were compared by performing an unpaired Student’s t test with Welch’s correction. Correlation analysis between discrete variables was assessed using Pearson’s coefficient. Data are presented as individual data plots with the mean of the individual group. Statistical analysis was performed using GraphPad Prism 6.0 software (GraphPad, La Jolla, CA, USA). Differences in RNA abundance with a *p* value <0.05 were considered significant.

## 3. Results

### 3.1. Subpopulations of Extracellular Vesicles Depend on the Isolation Method

**Figure 1:**
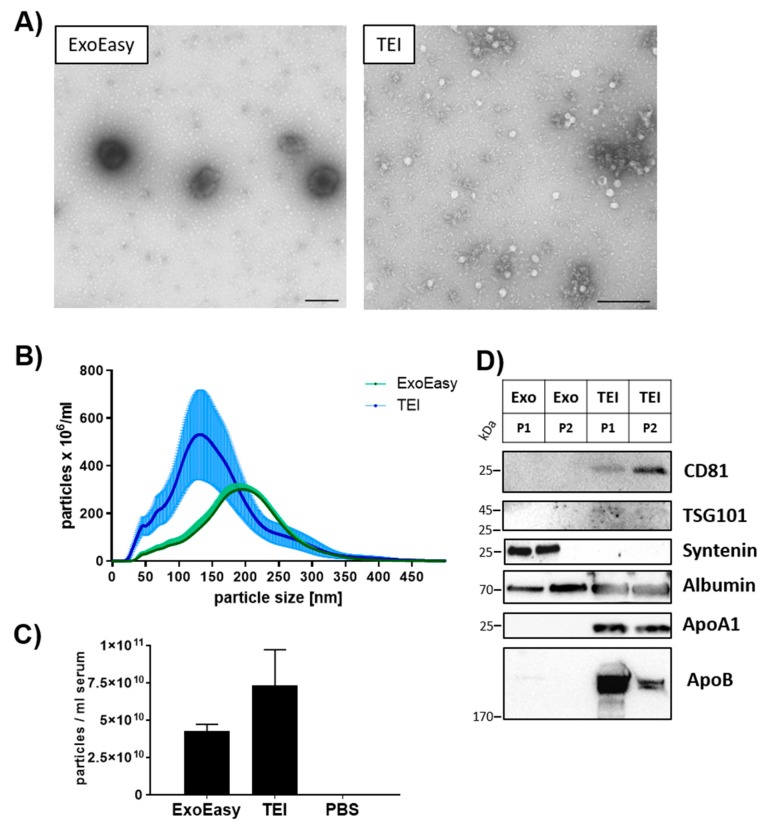
Characterization of extracellular vesicles. EVs were isolated by either membrane affinity (ExoEasy) or precipitation (TEI) methods. (**A**) Transmission electron microscopy was utilized to analyze morphology of isolated particles; scale bar = 200 nm. (**B**) The size profile of particles was assessed by nanoparticle tracking analysis (NTA) (mean and SEM of n = 4 individuals). (**C**) The total amount of particles from NTA in (**B**) was calculated. (**D**) Western blot analysis determined the presence of EV-associated markers and contamination with lipoproteins and soluble proteins. Serum from two healthy individuals (P1, P2) was used for EV isolation. A total of 20 µg total protein was loaded onto the gel.

For the isolation and enrichment of EVs from patients’ serum, we used two different isolation methods: a membrane affinity-based method (ExoEasy), and a precipitation-based method (TEI). We observed clear differences in the EV subpopulations isolated by the two methods (Figure 1). Transmission electron microscopy (TEM) analysis depicted round-shaped EVs in both isolates and a lipid double layer in the ExoEasy isolates (Figure 1A,B). The size of the EVs was measured by nanoparticle tracking analysis. The abundance of EVs isolated by ExoEasy peaked at about 200 nm in diameter, whereas EVs isolated by TEI represented a subpopulation of 50–200 nm with a peak at about 150 nm (Figure 1B). Furthermore, TEI isolation resulted in higher numbers of particles/mL serum than the ExoEasy protocol (7.5 × 10^10^ vs. 4 × 10^10^ particles/mL serum, mean, n = 4) (Figure 1C).

To characterize the EV subpopulation in more depth, we performed biochemical analysis of EV isolates (Figure 1D). We observed that the TEI isolates were enriched in EVs containing CD81 and TSG101, whereas the ExoEasy isolates were positive for the cytosolic protein Syntenin, which has been described as a marker for “smallEVs” [30]. Thus, we conclude that the two methods result in the isolation of different EV subpopulations.

Previous studies of body fluids suggested that the identified sncRNAs, which were claimed to be EV associated, might also be EV independent and lipid associated instead. Therefore, we analyzed the lipoprotein content of the isolates [31,32]. Lipoprotein contamination was demonstrated by detection of ApoA1 and ApoB, which were identified in TEI isolates (Figure 1D). In contrast, the amount of these two markers was much lower in ExoEasy isolates. Albumin was detected in isolates independent of the method used.

In summary, our data suggest that the TEI method enriches small EV subpopulations positive for the exosome-enriched protein CD81 without reducing contaminants such as lipoproteins or soluble proteins. In contrast, the ExoEasy method selects for syntenin-positive “small EVs” and appears to include larger EV subtypes. Importantly, ExoEasy reduces lipoprotein contaminants effectively.

### 3.2. Identification of Novel miRNA-Based Biomarkers for CTEPH by RNA Sequencing

**Figure 2:**
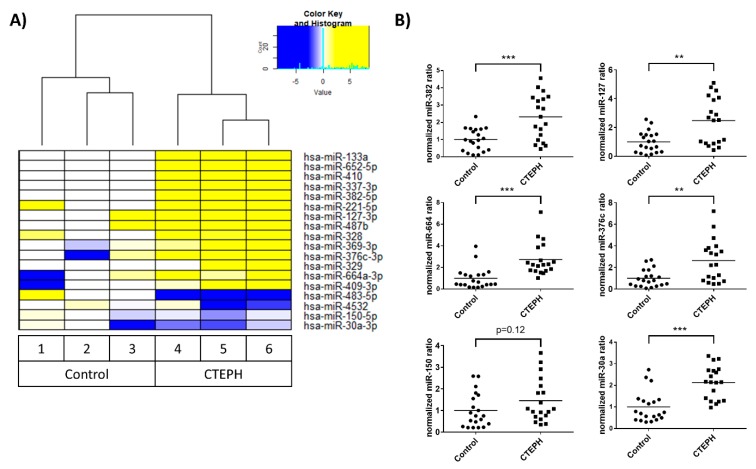
Differentially regulated EV-associated miRNAs in chronic thromboembolic pulmonary hypertension (CTEPH) vs. control individuals. EV- associated miRNAs were isolated using the exoRNeasy protocol. (**A**) RNA sequencing (n = 3) was performed using TrueQuant technology followed by sequencing. Depicted is the log_2_ (fold change) of the samples compared with the median of the controls. (**B**) miRNA abundance was standardized to the spike-in control cel-miR-39 and compared with that of healthy controls. The mean of the individual groups is depicted as a line. Significance was calculated using Student’s *t*-test with Welch’s correction. * *p* < 0.05, ** *p* < 0.01, *** *p* < 0.001.

The major challenge in identifying novel RNA-based biomarkers is the isolation of EVs from small-scale liquid biological samples. For RNA sequencing, three serum samples each from patients with CTEPH and control individuals were processed (Table 1). By applying the ExoEasy protocol, we were able to isolate a sufficient amount of RNA from as little as 100 µL serum starting volume. We made use of TrueQuant technology (GenXPro, Frankfurt am Main, Germany). Thus, a small RNA library was prepared from serum samples to identify a global profile of sncRNAs. TrueQuant Technology eliminates PCR-derived reads from the gene expression profiles generated [33]. We identified 18 miRNAs that were differentially regulated in CTEPH versus control individuals (Figure 2A).

For validation of the RNA sequencing, we isolated EVs from 300 µL serum of CTEPH patients (n = 20) and control individuals (n = 20) (Table 1) using the ExoEasy protocol. We analyzed six out of the 18 differentially regulated miRNAs by qRT-PCR and observed that four out of six miRNAs analyzed (miR-382, miR-127, miR-664, and miR-376c) were significantly upregulated in the CTEPH validation cohort compared with the control group (Figure 2B).

### 3.3. EV-Mediated miRNA Profile Depends on the EV Isolation Method

**Figure 3:**
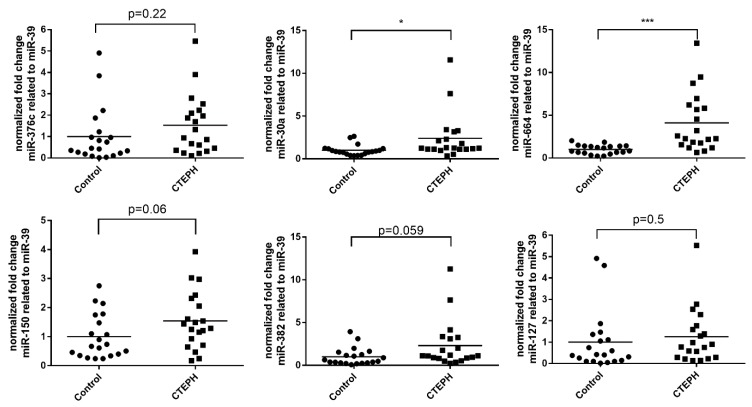
EV-associated miRNA abundance in serum of CTEPH patients (n = 20) and healthy controls (n = 20). EVs were isolated by the total exosome isolation (TEI) method and associated miRNAs were isolated using the ExoEasy protocol. miRNA abundance was standardized to the spike-in control cel-miR-39 and compared with that of control individuals. Lines represent mean values. Significance was calculated using Student’s t-test with Welch’s correction. * *p* < 0.05, ** *p* < 0.01, *** *p* < 0.001.

To investigate whether these observations are dependent on the isolated EV subpopulations, we compared the abundance of EV-associated miRNAs in TEI and ExoEasy isolates obtained from the serum of healthy individuals and CTEPH patients. We observed that miR-30a and miR-664a were significantly upregulated in EVs from CTEPH patients independent of the isolation method (compare Figure 3 with Figure 2). However, a direct comparison of the two methods shows that the abundance of miR-127 and miR-376c differs markedly in certain patients between the two methods (Suppl. Figure S1).

### 3.4. Identification of piRNAs as Novel Diagnostic Biomarkers for CTEPH

**Figure 4:**
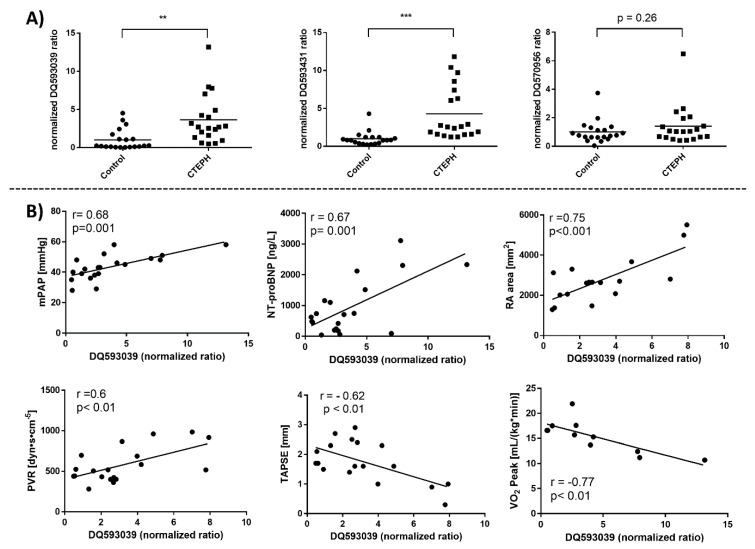
Differentially regulated EV-associated piRNAs in CTEPH patients vs. control individuals. EV-associated piRNAs were isolated by using the ExoEasy protocol. (**A**) qRT-PCR was performed to validate findings of the RNA sequencing. piRNA expression was standardized to the spike-in control cel-miR-39 and compared with that of controls. Lines represent mean values. Significance was determined using Student’s *t*-test with Welch’s correction. * *p* < 0.05, **p < 0.01, *** *p* < 0.001; n = 20. (**B**) The clinical parameters mean pulmonary arterial pressure (mPAP), pulmonary vascular resistance, NT-proBNP levels, TAPSE, RA area, and VO_2_ peak were compared with the abundance of EV- associated piRNAs in CTEPH patients by correlation analysis. Pearson correlation with 95% confidence interval is shown for normalized ratios of the EV-associated piRNA QD593039 compared with the individual clinical parameter.

The RNA screening approach mapped a number of sncRNA classes. piRNAs measured in EVs isolated by the ExoEasy protocol accounted for 13.3% of all sncRNAs mapped in the CTEPH group, whereas piRNAs made up 11.9% of non-coding RNAs in the control group, constituting the major subpopulation of sncRNAs mapped in the samples next to the miRNAs (Suppl. Figure S2A). We identified 21 differentially abundant piRNAs/piRNA clusters. All piRNAs are named in accordance with the NCBI accession number. One piRNA in particular, DQ593039, was highly upregulated in EVs from CTEPH patients, whereas various piRNA-clusters were downregulated (Suppl. Table S1). The upregulation of EV-associated DQ593039 in CTEPH patients was confirmed by qRT-PCR analysis (n = 20 individuals per group) (Figure 4A). The piRNA DQ593431 was upregulated in EVs from CTEPH patients (Figure 4B), whereas clusters comprising DQ593431 (e.g., DQ593431 + DQ597110) were downregulated in CTEPH patients demonstrated by RNA sequencing (Suppl. Table S1). DQ570956-containing clusters were downregulated in the three CTEPH patients analyzed by RNA sequencing (Suppl. Table S1), and levels of EV-associated DQ570956 in the validation study were similarly abundant in CTEPH and control individuals (n = 20) (Figure 4A).

In order to identify the potential of the differentially regulated EV-associated piRNAs as biomarkers for CTEPH, we used Pearson correlation analysis to link their abundance to clinically meaningful parameters defining CTEPH. We observed an association between DQ570956 and pulmonary vascular resistance (PVR) (r = 0.53, *p* <0.02, n = 20) but no correlation with any other clinical parameter (data not shown). Importantly, we observed a significant correlation between the abundance of EV-associated DQ593039 and mPAP (r = 0.68), PVR (r = 0.6), levels of N-terminal pro- brain natriuretic peptide (NT-proBNP) (r = 0.67), right atrium area (RA area) (r = 0.75), tricuspid annular plane systolic excursion (TAPSE) (r = −0.62), and peak oxygen uptake (VO_2_ peak) (r = −0.77) (Figure 4B).

### 3.5. Predicted Functional Role of piRNAs

To understand the role of the identified piRNAs in CTEPH progression, we predicted their putative mRNA targets via miRanda [23], as proposed by Hashim et al. [24]. Using an alignment score of 190, we identified 152 gene targets of the differentially regulated piRNAs. Gene ontology (GO) classification using the DAVID functional annotation tool [25] predicted a regulatory role of the identified piRNAs in various signaling pathways (Table 2), whereas four target genes (ADAMTS6, NDST1, DNM2, and SNX17) (Suppl. Table S2) contributed to the three most significant pathways: aorta development, coronary vascular development, and cardiac septum development (Table 2).

## 4. Discussion

Here we document for the first time the differential abundance of piRNAs in EVs of PH patients versus those from control individuals and identify piRNA DQ593039 as a promising biomarker reflecting the severity of the disease. Furthermore, based on their sequence we were able to predict the contribution of piRNAs to signaling pathways that might be involved in disease progression. Although the transport of miRNAs in EVs is well documented in various pulmonary diseases [34], the identification of specific EV-associated sncRNA profiles has not yet been described for patients with PH, or more precisely CTEPH. Here, we used serum samples of patients with diagnosed CTEPH.

Several protocols for the isolation of EVs have been developed in the last few years [35,36,37,38,39]. For clinical diagnostics and therapeutic approaches, standardized and commercial methods are of high interest. Recent publications have demonstrated that isolation methods based on membrane affinity or on precipitation are most suitable for miRNA biomarker discovery [40]. We made use of two different commercially available methods for the isolation and purification of EVs and their associated sncRNAs. ExoEasy and TEI resulted in the identification of differentially abundant EV- associated sncRNA profiles in CTEPH versus control samples. Even though Bland–Altman analysis displayed high similarity between the two methods (Suppl. Figure S1), only two out of five significantly upregulated miRNAs in ExoEasy isolates were confirmed by the TEI protocol (Figure 3). Differences in the expression profiles can be partly explained by the differences in EV subpopulations enriched in the isolates, which has been previously well documented [35,36]. Importantly, future studies have to consider that the choice of the EV extraction method will lead to tremendous differences in EV subpopulations that are enriched and thus to differences on EV- associated sncRNA profiles identified. Future studies will have to discover the EV subtype that shows the most relevant disease specific sncRNA profiles.

The method used for the isolation of EVs defines the purity of the EV isolate and the EV subpopulations that are enriched in the mixture [32,35,41]. The EV subpopulations we isolated differed in size and biochemical parameters (Figure 1). Our observations are consistent with the results reported by others [40]. In addition, we demonstrated that in contrast to TEI, the ExoEasy isolation protocol reduced lipoprotein contamination more efficiently (Figure 1D). Lipoproteins such as HDL and LDL were shown to transport sncRNA [42,43], so it is important to reduce lipoprotein contamination in order to ensure that the sncRNA profiles identified are related to EVs and not to lipoproteins. Based on our biochemical analysis, we assume that the sncRNAs detected in ExoEasy isolates are primarily EV associated. MiRNAs isolated by both procedures may attach to the surface of EVs or co-isolate with EVs (as expressed by the term “EV-associated”), which would not alter our conclusions in principle. Finally, using the membrane affinity method we were able to perform transcriptomic profiling of EV-associated sncRNAs from a mere 100 µL of serum, which is important considering that blood sample volumes are usually very limited.

While piRNAs are the most abundant sncRNA species in mammals, they are the least studied [44]. Rajan and colleagues showed the potential of piRNAs as diagnostic biomarkers [44]. They identified piRNAs in hypertrophied rat hearts that were differentially expressed compared with healthy control hearts and documented the differential abundance of a single piRNA, piR_2106027, in serum samples of patients with myocardial infarction versus controls. In the current study, we observed the differential abundance of various piRNAs associated with EVs in serum of CTEPH patients compared with control individuals. The piRNA DQ593039 was significantly upregulated in the CTEPH patients (n = 3) as determined by RNA sequencing and confirmed by qRT-PCR (n = 20) (Suppl. Table S1, Figure 4). Using miRanda we predicted that DQ593039 regulates the expression of the adaptor protein sorting nexin 17 (SNX17), which has been described to produce anti-arrhythmic effects by preserving functional SERCA2a protein in myocardial infarction [45]. This is in line with experimental studies on rats with right ventricular failure after chronic pulmonary artery banding that showed reduced SERCA2 mRNA expression levels in the right ventricle [46]. The significant correlation of DQ593039 levels with clinically meaningful parameters (mPAP, PVR, NT-proBNP, TAPSE, RA area, and VO_2_ peak; Figure 4B) suggests a pathological role of DQ593039 that is directly linked to the increased pressure in the pulmonary vasculature and the resulting changes in the right ventricle. This assumption is supported by our findings from target prediction and signaling pathway analysis that are consistent with the differentially regulated piRNAs being involved in processes of cardiac septum, and aortic and vascular development (Table 2).

Diagnosing CTEPH is challenging in the clinical routine. EV-associated DQ593039 correlates significantly with parameters that are relevant for diagnosis of patients with CTEPH, such as mPAP. Consequently, this piRNA could facilitate the decision whether obtaining further invasive diagnostics by right heart catheterization is necessary. Recently the European Society of Cardiology/European Respiratory Society (ESC/ERS) risk stratification scheme was applied to a real-life CTEPH cohort [47]. This scheme includes parameters such as TAPSE, RA area, and VO_2_ peak. These parameters, however, are not easy to determine, since they require well-trained examiners who are able to perform an echocardiographic examination according to the current guidelines. Additionally, cooperation of the patients is indispensable for parameters such as the VO_2_ peak that are obtained via spiro-ergometry. PVR is a good independent prognostic marker for CTEPH. Nevertheless, PVR can only be determined via invasive right heart catheterization, which is associated with a risk of severe complications. Thus, a new serum biomarker such as the EV-associated DQ593039 would make it easier to evaluate patients in the clinical setting.

Future studies involving larger numbers of patients are required to define a specific cut-off level to strengthen the proposed use of EV-associated DQ593039 as a biomarker for PH in clinical practice. In addition, investigating the expression level of EV-associated DQ593039 in other PH cohorts would confirm whether EV-associated piRNAs might be useful as biomarkers differentiating the various causes of PH.

## 5. Conclusions

In conclusion, we were able to identify specific profiles of EV-associated miRNAs and piRNAs in CTEPH patients that were different from those in control individuals. The upregulation of piRNA DQ593039 in EVs from CTEPH patients and its correlation with clinical parameters shows that this piRNA species may be useful as a biomarker for lung and heart diseases. Furthermore, we showed that the EV-associated sncRNAs identified here might play a role in disease progression. This finding might drive the development of pulmonary and cardiovascular therapeutics to promote lung and heart health.

## Figures and Tables

**Table 1:** Clinical characteristics of subjects.

Characteristics	RNA Sequencing		qRT-PCR	
	CTEPH (n = 3)	Controls (n = 3)	CTEPH (n = 20)	Controls (n = 20)
Sex (Male/total)	3/3	3/3	20/20	20/20
Age (yr)	67 (5)	67 (7)	60 (11)	56 (12)
PE (positive/total)	3/3	-	17/17	-
WHO class */NYHA *	2 (0)	1 (0.8)	2.5 (0.6)	1.2 (0.9)
6-MWD (m) *^‖^	432	-	519 (84)	-
MBP (mmHg) *	108 (20)	104 (7)	94 (13)	96 (9)
mPAP (mmHg) *	47 (4)	-	43 (9)	-
PVR (dyn s cm^−5^) *	465 (76)	-	551 (223)	-
CI (L min^−1^ m^−2^) *^$^	2.7 (0.5)	-	2.6 (0.6)	-
BMI	28 (5)	28 (3)	27 (4)	27 (4)
Arterial hypertension	1/3	2/3	5/20	12/20
Smoker	0/3	0/3	2/20 (7/20 formerly)	0/19 (4/19 formerly)
Coronary heart disease	0/3	0/3	1/19	0/20
Thrombophilia	0/3	-	3/18	-
Riociguat or PAH-medication	0/3	-	6/20	-
LVEF (%) *^$‡^	63 (9)	64 (2)	60 (6)	64 (7)
TAPSE (cm) *^$^	1.3 (0.4)	-	1.6 (0.7)	-
NT-proBNP (ng/L) *^‖†∫^	279	-	950 (911)	28 (43)
Leucocytes *	7.2 (1.5)	6.4 (1)	6.7 (2.1)	2.8 (5.5)
eGFR (mL/min/1.72 m^2^) *^§^	85 (15)	94 (5)	87 (21)	106 (19)

Definition abbreviations: CTEPH, chronic thromboembolic pulmonary hypertension; qRT-PCR, quantitative real-time polymerase chain reaction; PE, pulmonary embolism; MBP, mean blood pressure; mPAP, mean pulmonary arterial pressure; PVR, pulmonary vascular resistance; CI cardiac index; 6-MWD, 6-min walking distance; WHO, World Health Organisation; NYHA, New York Heart Association; BMI, body-mass index; PAH, pulmonary arterial hypertension; TAPSE, tricuspid annular plane systolic excursion; LVEF, left ventricular ejection fraction; NT-proBNP, N-terminal pro-brain natriuretic peptide; eGFR, estimated glomerular filtration rate; WHO class for CTEPH patients and Dyspnoea (NYHA) for controls depicted; * Mean (SD); for RNA-sequencing cohort; ^‖^ n = 1; for qRT-PCR; CTEPH; ^$^ n = 18; ^†^ n = 13; control; ^§^ n = 19 ^‡^ n = 14; ^∫^ n = 10; ^‖^ n = 5.

**Table 2:** Signaling pathways identified from biological process gene ontology (GO) enrichment using DAVID based on targeted genes predicted using miRanda software.

Term	Count	% Targeted Genes	*p*-Value	List Total	Pop Hits	Pop Total	Fold Enrichment	Bonfer-roni	Benja-mini	False Discovery Rate (FDR)
GO:0035904~aorta development	4	2.857	0.0002	110	18	16,792	33.92	0.13	0.13	0.30
GO:0060976~coronary vasculature development	4	2.857	0.0006	110	25	16,792	24.42	0.31	0.17	0.82
GO:0003279~cardiac septum development	3	2.143	0.0026	110	12	16,792	38.16	0.83	0.45	3.89
GO:0000301~retrograde transport, vesicle recycling within Golgi	2	1.429	0.0383	110	6	16,792	50.88	1.00	1.00	44.33
GO:0048812~neuron projection morphogenesis	3	2.143	0.0388	110	48	16,792	9.54	1.00	0.99	44.75
GO:1901796~regulation of signal transduction by p53 class mediator	4	2.857	0.0469	110	124	16,792	4.92	1.00	1.00	51.34
GO:0006355~regulation of transcription, DNA-templated	16	11.429	0.0618	110	1504	16,792	1.62	1.00	1.00	61.57
GO:0055085~transmembrane transport	5	3.571	0.0746	110	244	16,792	3.13	1.00	1.00	68.70
GO:0030154~cell differentiation	7	5.000	0.0803	110	462	16,792	2.31	1.00	1.00	71.50
GO:0061029~eyelid development in camera-type eye	2	1.429	0.0812	110	13	16,792	23.49	1.00	1.00	71.90
GO:0006888~ER to Golgi vesicle-mediated transport	4	2.857	0.0860	110	160	16,792	3.82	1.00	1.00	74.04
GO:0016578~histone deubiquitination	2	1.429	0.0872	110	14	16,792	21.81	1.00	0.99	74.52
GO:0006816~calcium ion transport	3	2.143	0.0873	110	76	16,792	6.03	1.00	0.99	74.59
GO:0035994~response to muscle stretch	2	1.429	0.0990	110	16	16,792	19.08	1.00	0.99	79.04

## Supplementary Materials

The following are available online at https://www.mdpi.com/2218-273X/9/11/666/s1, Figure S1: Comparison of the EV-associated miRNA expression levels of TEI and ExoRNeasy protocol isolates., Figure S2: Small RNA profiles and mapping statistics of EV-RNA from CTEPH patients and control individuals Table S1: Normalized expression levels of differentially expressed piRNA clusters, Table S2: Selection of predicted target genes of the differentially regulated piRNAs, Table S3: piRNA sequences used for TaqMan Assay design, Table S4: TaqMan MicroRNA assays (ThermoFisher scientific, Germany) for RT-PCR analysis.
