# Supplementary material for: Non-Invasive Approach for Evaluation of Pulmonary Hypertension Using Extracellular Vesicle-Associated Small Non-Coding RNA
Source: Biomolecules. 2019 Oct 29;9(11):666. doi: 10.3390/biom9110666 (PMC6920761; doi:10.3390/biom9110666)
Supplement: Supplementary file 1 [file biomolecules-09-00666-s001.pdf]

Supplementary material

Non-invasive approach for evaluation of pulmonary hypertension using extracellular vesicle-associated small non-coding RNA

Christoph Lipps [1,2,3,*], Philipp Northe [1,3], Ricardo Figueiredo [4], Manfred Rohde [5], Alexandra Brahmer [6], Eva-Maria Krämer-Albers [6], Christoph Liebetrau [2,3], Christoph B. Wiedenroth [7], Eckhard Mayer [7], Steffen D. Kriechbaum [2,3], Oliver Dörr [1, 2,3], Holger Nef [1, 2,3], Christian W. Hamm [1, 2,3], Till Keller [1, 2,3] and Christian Troidl [1,2,3]

[1] Medical Clinics I-Cardiology and Angiology, Justus-Liebig-University Giessen, 35392 Giessen, Germany;
[2] Department of Cardiology, Kerckhoff Clinic GmbH, 61231 Bad Nauheim, Germany;
[3] German Centre for Cardiovascular Research, Partner Site Rhine-Main, Benekestrasse 2–8, 61231 Bad Nauheim, Germany
[4] GenXPro GmbH, 60438 Frankfurt am Main, Germany;
[5] Central Facility for Microscopy, Helmholtz Centre for Infection Research, 38124 Braunschweig, Germany;
[6] Institute of Developmental Biology and Neurobiology, Biology of Extracellular Vesicles, Johannes Gutenberg-University, 55122 Mainz, Germany;
[7] Department of Thoracic Surgery, Kerckhoff Clinic GmbH, 61231 Bad Nauheim, Germany;

* Correspondence: christoph.lipps@innere.med.uni-giessen.de; Tel.: +49-641-99-42248

Supplementary figures

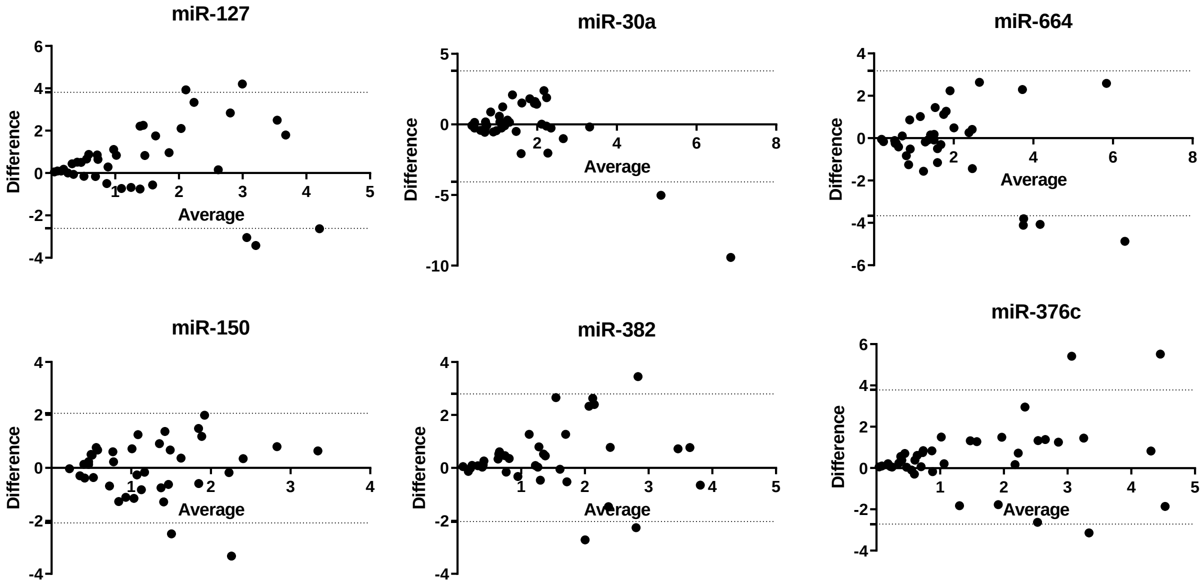

Suppl. Fig. S1: Comparison of the EV-associated miRNA expression levels of TEI and ExoRNeasy protocol isolates. Bland-Altman analysis with 95% confidence interval was performed for the normalized miRNA ratios of EVs isolated via the two protocols. Each dot represents an individual sample. Displayed is the difference (ExoEasy$_{normalized_ratio}$ - TEI$_{normalized_ratio}$) as a function of the average of ExoEasy$_{normalized_ratio}$ and TEI$_{normalized_ratio}$ for each individual sample.

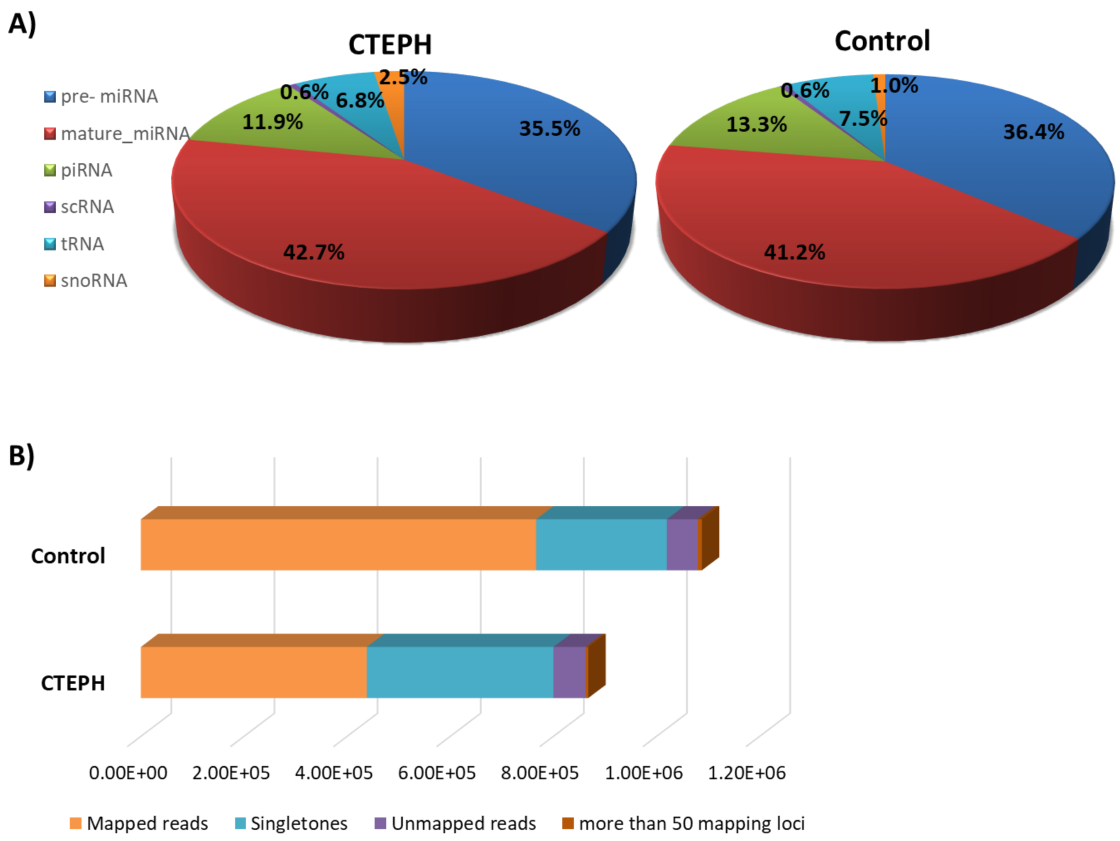

Suppl. Fig. S2: Small RNA profiles and mapping statistics of EV-RNA from CTEPH patients and control individuals. Top panel (A) shows the relative abundance of the reads annotated for the indicated RNA subclasses after normalization (pre-miRNA, mature miRNA, piRNA, small cytoplasmatic RNA (scRNA), transfer RNA (tRNA) and small nucleolar ribonucleic acid RNA (snoRNA). Bottom panel (B) shows the mapping statistics of the small RNA-seq data.

Supplementary tables

Suppl. Table 1: Normalized expression levels of differentially expressed piRNA clusters. Analysis of piRNAs isolated by ExoRNeasy using TrueQuant technology.

piRNA ID	CTEPH (Normalized expression)	Control (Normalized expression)	Log_2FC	p-value
hsa_piR_020500|gb|DQ598190	251.81	1102.68	-2.131	1.28E-05
hsa_piR_020500|gb|DQ598190; hsa_piR_020793|gb|DQ598619	61.57	272.036	-2.144	2.56E-05
hsa_piR_020500|gb|DQ598190; hsa_piR_020497|gb|DQ598177	64.50	278.84	-2.112	3.42E-05
hsa_piR_015249|gb|DQ590830	14.92	307.643	-4.366	0.00012
hsa_piR_015026|gb|DQ590548	120.98	312.891	-1.371	0.00247
hsa_piR_020326|gb|DQ597916; hsa_piR_001311|gb|DQ571812; hsa_piR_018569|gb|DQ595533; hsa_piR_015026|gb|DQ590548	6941.61	17150.4	-1.305	0.0026
hsa_piR_015026|gb|DQ590548; hsa_piR_018569|gb|DQ595533; hsa_piR_001311|gb|DQ571812; hsa_piR_020326|gb|DQ597916	3470.60	8574.83	-1.305	0.00261
hsa_piR_000765|gb|DQ570956; hsa_piR_001312|gb|DQ571813; hsa_piR_018570|gb|DQ595536	3485.47	8588.33	-1.301	0.00269
hsa_piR_018570|gb|DQ595536; hsa_piR_001312|gb|DQ571813; hsa_piR_000765|gb|DQ570956	14295.50	34964.4	-1.290	0.00303
hsa_piR_009295|gb|DQ582567; hsa_piR_001312|gb|DQ571813; hsa_piR_018570|gb|DQ595536	3729.58	9090.37	-1.285	0.00328
hsa_piR_018570|gb|DQ595536; hsa_piR_001312|gb|DQ571813; hsa_piR_000765|gb|DQ570956; hsa_piR_000291|gb|DQ570344	3641.22	8869.98	-1.285	0.00332
hsa_piR_018570|gb|DQ595536; hsa_piR_001312|gb|DQ571813; hsa_piR_020326|gb|DQ597916	3641.46	8869.17	-1.284	0.00333
hsa_piR_020326|gb|DQ597916; hsa_piR_001312|gb|DQ571813; hsa_piR_018570|gb|DQ595536	3641.87	8870.87	-1.284	0.00333
hsa_piR_018569|gb|DQ595533; hsa_piR_015026|gb|DQ590548	337.71	818.684	-1.278	0.00394
hsa_piR_000794|gb|DQ570992; hsa_piR_019914|gb|DQ597347; hsa_piR_016945|gb|DQ593356; hsa_piR_022628|gb|DQ600952	85.39	267.591	-1.648	0.00945
hsa_piR_022628|gb|DQ600952; hsa_piR_016945|gb|DQ593356; hsa_piR_019914|gb|DQ597347; hsa_piR_016742|gb|DQ593049	169.66	487.321	-1.522	0.01322
hsa_piR_019752|gb|DQ597110; hsa_piR_016926|gb|DQ593325; hsa_piR_016984|gb|DQ593431	4311.78	49964.3	-3.535	0.02069
hsa_piR_016984|gb|DQ593431; hsa_piR_016926|gb|DQ593325; hsa_piR_019752|gb|DQ597110	7370.39	83776.8	-3.507	0.02115
hsa_piR_016984|gb|DQ593431; hsa_piR_019752|gb|DQ597110	1474.10	16737.6	-3.505	0.02121
hsa_piR_019914|gb|DQ597347; hsa_piR_016742|gb|DQ593049; hsa_piR_016945|gb|DQ593356; hsa_piR_022628|gb|DQ600952	169.85	440.224	-1.374	0.02379
hsa_piR_016735|gb|DQ593039	179.97	15.6378	3.525	0.03029
hsa_piR_017724|gb|DQ594465; hsa_piR_004308|gb|DQ575882; hsa_piR_016745|gb|DQ593052	403.73	942.526	-1.223	0.03103
hsa_piR_004308|gb|DQ575882; hsa_piR_017724|gb|DQ594465; hsa_piR_016745|gb|DQ593052	590.13	1341.65	-1.185	0.03302

Suppl. Table 2: Selection of predicted target genes of the differentially regulated piRNAs. MiRanda software with a minimum score of 190 was used for target prediction.

Gene symbol	piRNA	Targeted transcript ID	Tot Score	Tot Energy	Max Score	Max Energy	Strand	Len1	Len2	Positions
ADAMTS6	DQ593431	ENST00000381055.7	190.00	-37.66	190.00	-37.66	124442	32	7311	6531
	DQ593431	ENST00000381052.8	190.00	-37.66	190.00	-37.66	124443	32	6956	6179
NDST1	DQ570956	ENST00000523767.5	193.00	-33.67	193.00	-33.67	1838333	31	3449	1666
	DQ597916	ENST00000523767.5	193.00	-32.79	193.00	-32.79	2200071	31	3449	1666
DNM2	DQ571813	ENST00000590806.5	194.00	-32.93	194.00	-32.93	1783006	31	5651	1188
	DQ571813	ENST00000590787.1	194.00	-32.93	194.00	-32.93	1783007	31	4299	499
SNX17	DQ593039	ENST00000233575.6	201.00	-36.78	201.00	-36.78	4455891	31	2044	29
	DQ593039	ENST00000537606.5	201.00	-36.78	201.00	-36.78	4455900	31	2400	55

Suppl. Table 3: piRNA sequences used for TaqMan Assay design

NCBI accession number	Sequence
DQ593431	CGTGCTGGGCCCATAACCCAGAGGTCGATGGA
DQ570956	AGCATTGGTGGTTCAGTGGTAGAATTCTCGC
DQ593039	CCGCCTGGGAATACCGGGTGCTGTAGGCTTA

Suppl. Table 4: TaqMan MicroRNA assays (ThermoFisher Scientific, Germany) for RT-PCR analysis

Target	*Assay ID*
hsa-miR-664a-3p	002897
hsa-miR-382-5p	000572
hsa-miR-127-3p	000452
hsa-miR-376c-3p	002122
hsa-miR-30a-5p	000417
hsa-miR-150-5p	000473
cel-miR-39	000200
